# Real-time quantification of bowel perfusion using Laparoscopic Laser Speckle Contrast Imaging (LSCI) in a porcine model

**DOI:** 10.1186/s12893-023-02161-w

**Published:** 2023-08-31

**Authors:** Saloni Mehrotra, Yao Z. Liu, Chibueze A. Nwaiwu, Vasiliy E. Buharin, Roman Stolyarov, Steven D. Schwaitzberg, Matthew F. Kalady, Peter C. W. Kim

**Affiliations:** 1 Department of Surgery, University at Buffalo, Buffalo, NY USA; 2 Activ Surgical Inc., Boston, MA USA; 3 Department of Surgery, Brown University, Providence, Rhode Island USA; 4 Department of Surgery, The Ohio State University Wexner Medical Center, Columbus, OH USA

**Keywords:** Bowel perfusion, Laser speckle contrast imaging, Perfusion quantification, Anastomotic leak, Indocyanine green

## Abstract

**Background/purpose:**

Real-time quantification of tissue perfusion can improve intraoperative surgical decision making. Here we demonstrate the utility of Laser Speckle Contrast Imaging as an intra-operative tool that quantifies real-time regional differences in intestinal perfusion and distinguishes ischemic changes resulting from arterial/venous obstruction.

**Methods:**

Porcine models (*n* = 3) consisted of selectively devascularized small bowel loops that were used to measure the perfusion responses under conditions of control/no vascular occlusion, arterial inflow occlusion, and venous outflow occlusion using laser speckle imaging and indocyanine green fluoroscopy. Laser Speckle was also used to assess perfusion differences between small bowel antimesenteric-antimesenteric and mesenteric-mesenteric anastomoses. Perfusion quantification was measured in relative perfusion units calculated from the laser speckle perfusion heatmap.

**Results:**

Laser Speckle distinguished between visually identified perfused, watershed, and ischemic intestinal segments with both color heatmap and quantification (*p* < .00001). It detected a continuous gradient of relative intestinal perfusion as a function of distance from the stapled ischemic bowel edge. Strong positive linear correlation between relative perfusion units and changes in mean arterial pressure resulting from both arterial (*R*^2^ = .96/.79) and venous pressure changes (*R*^2^ = .86/.96) was observed. Furthermore, Laser Speckle showed that the antimesenteric anastomosis had a higher perfusion than mesenteric anastomosis (*p* < 0.01).

**Conclusions:**

Laser Speckle Contrast Imaging provides objective, quantifiable tissue perfusion information in both color heatmap and relative numerical units. Laser Speckle can detect spatial/temporal differences in perfusion between antimesenteric and mesenteric borders of a bowel segment and precisely detect perfusion changes induced by progressive arterial/venous occlusions in real-time.

## Introduction

A number of critical factors including microbiome and perfusion are necessary for optimal tissue healing of wounds and anastomoses [1]. Poor anastomotic perfusion is believed to be associated with increased anastomotic leakage [2, 3], which in turn is associated with increased short- and long-term morbidity and mortality, prolonged hospital stays, and increased healthcare costs are attributed to anastomotic leaks [4]. Additionally, poor perfusion can also result in chronic ischemia at anastomosis, which may cause anastomotic strictures, requiring further endoscopic procedures or reoperations [5]. The ability to precisely and accurately determine tissue perfusion intra-operatively in real time may mitigate the physiological causes of anastomotic leaks and improve surgical outcomes.

Real-time tissue perfusion and viability at anastomotic sites have been assessed intraoperatively predominantly using visual and subjective intra-operative benchmarks such as mesenteric pulsation, coloration of tissue and bleeding from resection lines by the naked eye [6]. However, studies show that visual clinical risk assessment by surgeons has low accuracy in predicting anastomotic leakage in gastrointestinal surgery [7]. These tools are subjective and cannot be used in minimally invasive surgeries. There have been several studies that have investigated tissue perfusion using doppler ultrasound [8] and laser doppler flowmetry [9]. However, the lack of reliability and clinical ease of use have limited routine use for intraoperative assessment of tissue perfusion [10].

Indocyanine green fluorescence angiography (ICG-FA) is a modality that is increasingly gaining acceptance as an adjunct in intra-operative assessment of tissue perfusion in laparoscopic and open gastrointestinal surgery [11]. ICG-FA relies on an intravascular injection of indocyanine green (ICG), a non-toxic water-soluble dye that binds to lipoproteins. The fluorescent spectrum of ICG is in the range of near-infrared light and can be captured by a specialized camera [12]. However, since its plasma disappearance rate is high, there are wide deviations in the fluorescence signal and thus subjectivity in the visual assessment of ICG perfusion assessment [13]. The usability of ICG is thus limited by its pharmacokinetics, lack of standardized signal interpretation, false positives with repeated use.

Laser Speckle Contrast Imaging (LSCI) is a relatively new technology that holds promise for the detecting and visualization of intra-operative tissue perfusion in gastrointestinal surgery. LSCI measures tissue blood flow by capturing dynamic changes in the interference pattern of laser light scatter from moving particles (e.g., red blood cells) [14]. This speckle contrast pattern is then processed to form a perfusion colormap image that changes in real time. LSCI can detect perfusion in real time without the need for injecting a fluorophore and produces repeatable signals that are unaffected by previous assessments [15]. This study focused on usage of the device as would be done clinically, which we have demonstrated in pre-clinical and clinical settings to be reliable and repeatable [15–17]. We have previously demonstrated that ischemia identified by decreased perfusion on LSCI correlates with increased tissue capillary lactate levels within local tissue [18, 19].

In this study we introduce an investigational quantification feature of LSCI correlating the relative colors on the perfusion heatmap to a numerical perfusion metric. Using this feature, we aim to demonstrate to spatiotemporal accuracy of LSCI and its ability to distinguish detect real time physiologic changes and regional differences in bowel perfusion. LSCI with its prototype quantification feature may promise clinical utility for more objective perfusion assessment of gastrointestinal anastomoses.

## Materials and methods

### Combining Laser Speckle Contrast Imaging (LSCI) and ICG -FA technology

**Fig. 1:**
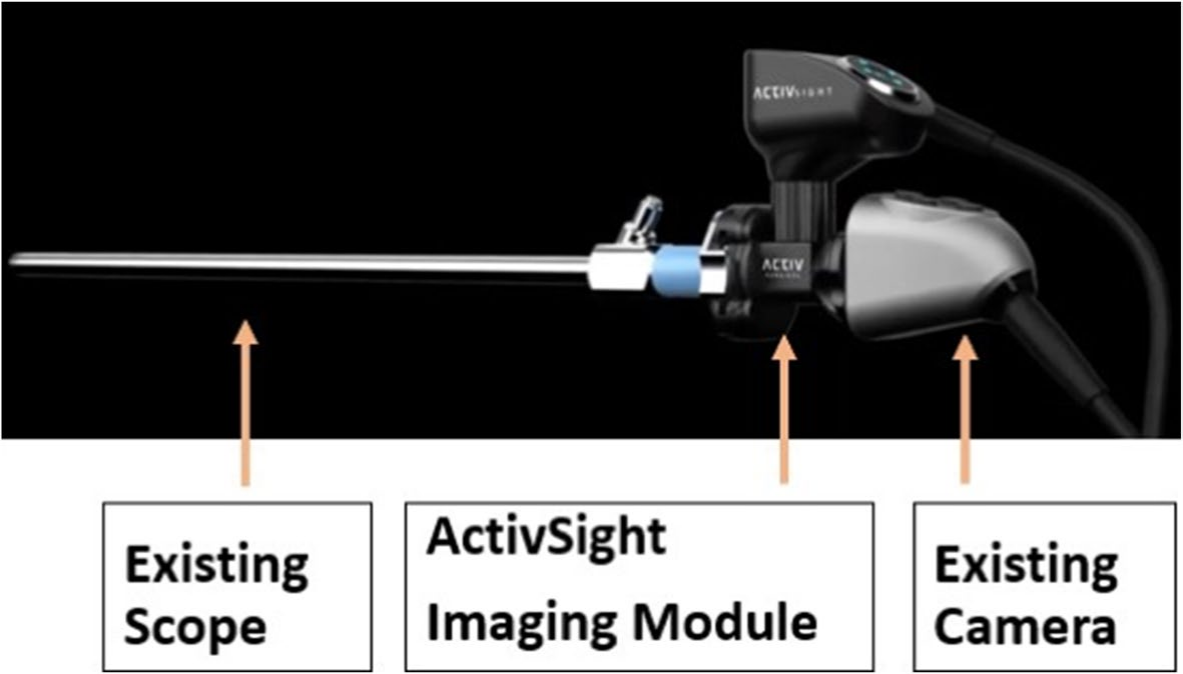
ActivSight™. The ActivSight™ imaging module is a device that is positioned between a standard white light camera head and a standard laparoscope

The device used in this study, ActivSight™ imaging module, is FDA-cleared to enable intraoperative surgical visualization. The device allows for visualization of LSCI as well as ICG fluorescence in a modular laparoscopic form factor that integrates between a standard white light laparoscopic camera and the laparoscope (Fig. 1). The module passes visible light through the white light camera while re-directing near infrared light to an in-built sensor. The light from sensor is then sent to a light engine which then provides near infrared illumination allowing for either ICG-FA or LSCI processing. The images from white light camera and the infrared camera are then combined using proprietary ActivSight LSCI algorithms [20] to create an overlay that shows augmented surgical video [15]. The module provides two different modes of data augmentation—i) ICG fluorescence angiography ii) LSCI generated perfusion color heatmap. Warmer colors (red, yellow) on the LSCI map indicate areas of higher blood flow/tissue perfusion while cooler colors (blue, green) represent areas of low blood flow/tissue perfusion.

ActivSight™ LSCI laser operates at the wavelength of 852 nm in the near-infrared spectrum. Laser output power is 50 milliwatts emitted from the scope. The image is then received and processed on a special high IR sensitivity sensor, with a pixel per speckle ratio of approximately 1:1 and a speckle framerate of 120fps. The system uses a temporal window chosen to smooth and improve image quality. To closely match the standard white light field of view, ActivSight™ is designed with a field of view of 60 degrees divergence, ± 30 degrees.

LSCI data from the target tissues were quantified using relative perfusion units (RPUs). This methodology converts raw LSCI data, which can differ in absolute value due to technical, equipment, patient, and tissue factors, into a relative scale. RPU quantifies LSCI perfusion using a relative scale bounded by values of maximally (“Hot”) and minimally (“Cold”) perfused areas of the target tissue. The Hot region represents the raw LSCI values of a surface level capillary on the target tissue, which tend to have the highest LSCI values. The Cold region represents another region associated with the lowest values within the ischemic area of the target tissue. RPU of regions of interest (ROIs) on the target tissue are calculated from raw LSCI values with the equation, ROI^RPU^ = (ROI^Raw^ – Cold^Raw^) / (Hot^Raw^—Cold^Raw^). RPUs represent perfusion as percentage with a range from 0% (equal to the Cold region) to 100% (equal to the Hot region). This type of relative perfusion analysis is similar to those performed for quantification of ICG-FA [21], RPU values allow for pooled analysis and additional precision when comparing the magnitude and distribution of tissue ischemia. All selected regions including ROIs and Hot and Cold references have an area of 317 pixels. The RPUs are quantified within a single frame, and then values of 5 representative frames are averaged together. In this study, Hot and Cold regions are manually and not algorithmically selected, which leads to some RPU values of ROIs to be rarely slightly below 0% or slightly above 100%. This quantification feature is not present in the FDA-cleared version of the ActivSightTM module.

### Preclinical experiment design

Three 3-month-old Yorkshire pigs weighing between 42–53 kg were used. All animals used in this experiment were managed according to ethical guidelines per *IACUC Approved Protocol (# B2021-32)*, institutional swine anesthesia standard operating protocol and the ARRIVE 2.0 guidelines [22]. No genetic modifications/prior procedures were performed on animals. The animals were fasted on the morning of the day of surgical procedures. Anesthesia was induced using telazol (6 mg/kg), ketamine (3 mg/kg) and xylazine (3 mg/kg) and maintained using 1–2% isoflurane. All pigs were euthanized at the end of surgery. No specific exclusion criteria were used to exclude animals and all animals underwent same surgical procedure detailed below.

Regional differences in bowel perfusion were then studied in the following clinically relevant porcine intestinal models – 1) Partially de-vascularized linear small bowel segment under conditions of i) no vascular occlusion/control (physiologic blood flow), ii) arterial inflow occlusion iii) venous outflow occlusion and 2) Small bowel anastomoses (both mesenteric-to-mesenteric and antimesenteric-to-antimesenteric). The primary metric of perfusion evaluation included 1) real time perfusion colormap display and 2) perfusion quantification measured in relative perfusion units (RPUs). All LSCI-based measurements were performed with the laparoscope mounted on a scope holder. The laparoscope was placed perpendicular to and 20 cm above target tissue. This was done to ensure that the entire model was included in the field of view to minimize any potential differences in assessment and standardize the distance and angle. The scope holder also reduces motion artifacts. We have previously published in preclinical and clinical studies using ActivSight at this distance, as well as both larger and smaller distances intra- and extra-corporeally. Our preclinical studies in porcine models have been both laparoscopic and open, at varying distances, and have translated well to intracorporeal and extracorporeal human usage at standard distances in the OR [15–17, 23, 24]. RPUs were calculated using computational processing on the LSCI generated perfusion heatmap images. Perfusion values were generated by averaging values over twenty-one pixels and five frames while taking the cardiac cycle into account. Mean arterial pressure (MAP) was continuously measured via femoral arterial line.

### Surgical preclinical models

#### Partially de-vascularized linear small bowel model

For the linear porcine small bowel model, a loop of small bowel was extracted and stapled at 12′0 clock position using a linear stapler. Once the bowel was stapled, the mesentery was de-vascularized using electrosurgical coagulation approximately 4 cm from the stapled bowel edge to create a progressive gradient of small bowel ischemia. The small bowel was then visually categorized into segments of perfused, watershed and ischemic bowel based on naked eye assessment for degrees of perfusion (as a function of distance from devascularized bowel edge). The small bowel perfusion gradient was then continuously measured using LSCI under three conditions 1) no vascular occlusion (control), 2) arterial inflow occlusion via proximal aorta clamping and 3) venous outflow occlusion via portal venous clamping. Regions of interest within the bowel segments and along the antimesenteric and mesenteric arc were selected for perfusion quantification. A standard dose of 0.1–0.3 mg/kg of ICG was administered and bowel was observed 30–40 s after injection, which corresponded with the time to qualitative maximum intensity. This dosage and timing is consistent with dosages administered in previously published studies [15, 23, 25].

#### Mesenteric and antimesenteric small bowel anastomoses

To evaluate differences in bowel perfusion not only in a linear model but also in clinically relevant anastomoses models, we selected commonly used antimesenteric and mesenteric anastomoses in small intestine. Two loops of small bowel were extracted, and a side-to-side anastomosis was created on the conventional antimesenteric-antimesenteric border using a linear stapler. This was then repeated, and a side-to-side anastomosis was created closer to the mesenteric-mesenteric border while preserving the blood supply to the bowel. Both mesenteric and antimesenteric border small bowel anastomoses were examined using LSCI perfusion quantification.

### Statistical analysis

Statistical analyses were performed using Excel. Descriptive data are presented as mean and standard deviation (SD). Continuous variables (Mean RPUs) follow a normal distribution and were analyzed using students t-tests and ANOVA tests. The level of statistically significance was set a priori at 0.05. The relationship between the RPU’s and MAPs of various bowel segments with arterial/venous occlusion was analyzed using R^2^ correlation coefficient.

## Results

### Differences in perfusion over progressively ischemic bowel loop

**Fig. 2:**
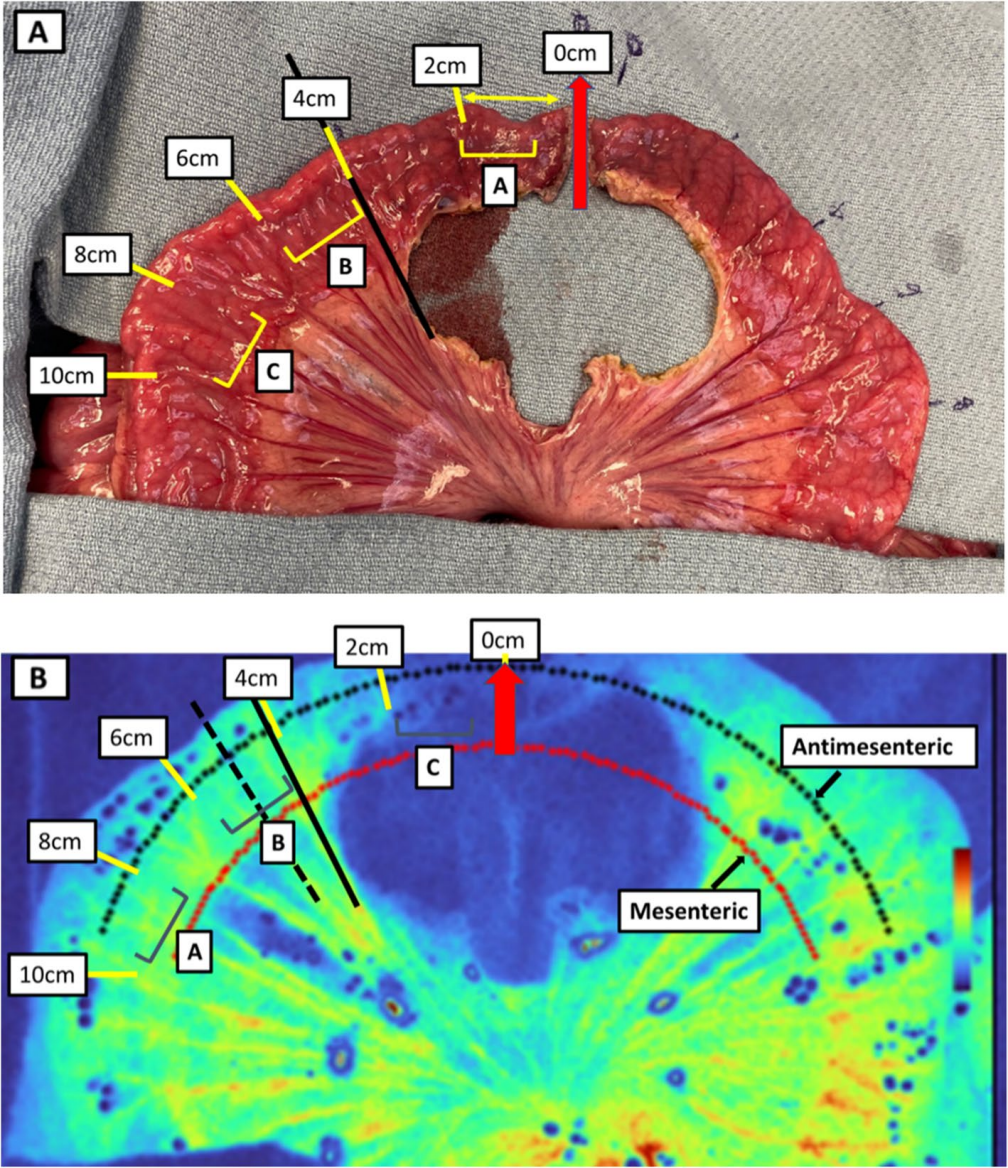
**a** RGB image of linear small bowel model experimental setup. The figure shows a randomly selected semi-circular loop of porcine small bowel bissected using stapler at 12′0 clock position (red arrow). This stapled bowel edge (marked 0 cm) was a benchmark to determine the extent of mesenteric devascularization. The bowel was then partially devascularized to approximately 4 cm from stapled bowel edge (solid black line) to create a perfusion gradient. Regions within this gradient were pre-labeled A = ischemic (0–2 from stapled edge), B = watershed (4–6 cm from stapled bowel) and C = perfused (8–10 cm from stapled bowel) based on naked eye estimates of bowel perfusion and the distance from the margin of devascularization. Bowel perfusion was then measured continuously along this gradient, according to radial distances from the stapled bowel edge (marked by yellow lines). **b** LSCI image of linear small bowel model experimental setup. The figure shows LSCI contrast images of the same semi-circular loop of porcine small bowel model stapled at 12′0 clock position (red arrow) and partially devascularized to approximately 4 cm from stapled bowel edge (solid black line) to create a perfusion gradient. In the color heatmap, warm colors (red, yellow) represent high perfusion while cool colors (blue) represent low perfusion. Regions within this gradient were pre-labeled A = ischemic, B = watershed and C = perfused based on naked eye estimates of bowel perfusion and the distance from the margin of devascularization. The three labelled bowel segments are represented by different colors on this heatmap. The ischemic bowel segment is dark blue, the watershed segment is green while the perfused segment is yellow/red. For quantitative assessment, RPUs are measured radially over the continous bowel gradient along the mesenteric (dotted red line) and antimesenteric (dotted black line) arcs. The dash black line represents the radial distance (5 cm from the stapled edge) from which the perfusion values start to decrease

In the ischemic bowel segment, the loop was then divided with a standard linear stapling device. The mesentery was then partially de-vascularized 4 cm from each stapled bowel edge to create a perfusion gradient as a function of distance. This small bowel loop was inspected and then categorized into pre-labeled segments of ischemic (0–2 from stapled edge), watershed (4–6 cm from stapled bowel) and perfused (8–10 cm from stapled bowel) bowel. This labeling was based on consensus reached by 3 clinicians using conventional visual inspection and distance relative to the margin of devascularization (Fig. 2a). The small bowel loop was then observed under LSCI mode to enable quantification of perfusion values (Fig. 2b).

**Fig. 3:**
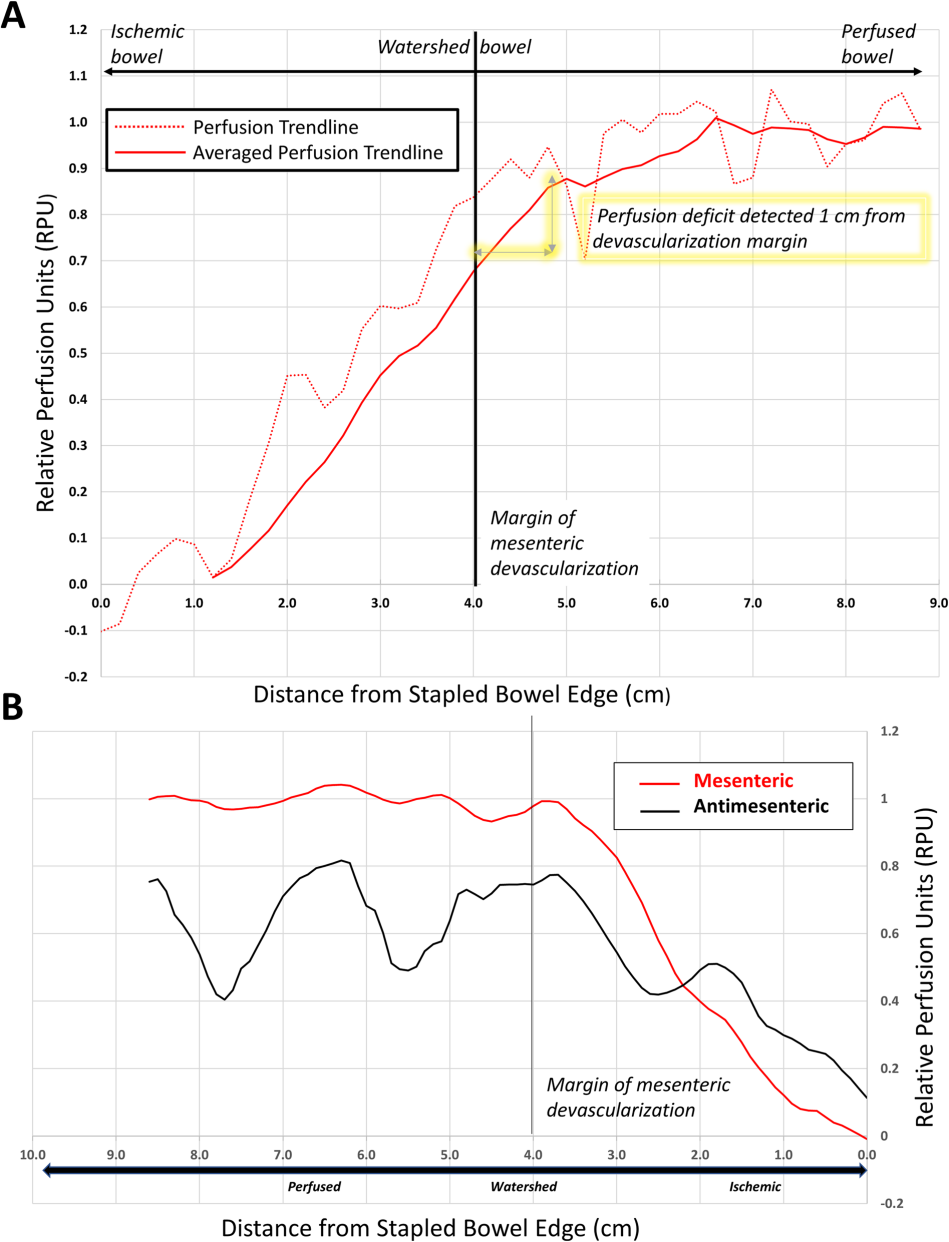
**a** Perfusion over ischemic bowel gradient as a function of distance from stapled bowel edge. Intestinal perfusion in Relative Perfusion Units (RPU) on Y-axis vs Radial distance from stapled bowel edge (cm) on X-axis are shown. The continuous bowel gradient is divided into ischemic, watershed and perfused bowel segments with increasing distance from stapled bowel edge (from left to right). In contrast to potentially subjective end user perception of color heatmap of perfusion, LSCI quantification detects a continuous gradient of corresponding numerical relative intestinal perfusion and clearly distinguishes between perfused, watershed, and ischemic segments using RPU quantification (*p* = 9 × 10–10). Perfusion values are found to decrease with deviating distance from stapled edge. There is a significant decrease in perfusion values noted at 1 cm from the margin of mesenteric devascularization. **b** Perfusion differences in antimesenteric & mesenteric bowel regions along ischemic bowel gradient. X-axis shows distance from the stapled bowel edge measured in cm; Y-axis shows LSCI-measured RPU of antimesenteric (black) and mesenteric (red) bowel regions. In perfused and watershed segments, the mean RPU of antimesenteric and mesenteric bowel was significantly different (*p* < 0.0001). Overall, for every distance along the perfused/watershed bowel segments, the bowel perfusion on the mesenteric border was significantly higher than the antimesenteric border

With LSCI, the labeled bowel segments were qualitatively differentiated by the concentration of colors on the heatmap over those regions—ischemic (dark blue), watershed (green), and perfused (yellow/red). Using RPU quantification metrics, LSCI could clearly distinguish between the pre-labeled perfused, watershed and ischemic segments of bowel (*p* < 0.00001). In this linear model, LSCI able to discern among categorically and visually pre-labeled regions, but it was also able to detect and display as hypothesized a continuous gradient of bowel perfusion as function of distance from the stapled bowel edge. Mean perfusion values declined from perfused (98.8% ± 5.0%) to watershed (78.3% ± 12.5%) to ischemic (1.9% ± 0.08%) bowel segments. The decline in perfusion values from the perfused (8–10 cm from stapled edge) to the watershed region (4–6 cm from stapled edge) was steady with no major deflection points. Interestingly, in this porcine model, LSCI detected a sharp decline in perfusion values starting at 5 cm from the stapled bowel edge (1 cm proximally from the de-vascularized mesenteric margin) (Fig. 3a). This steep decline continued to the ischemic segment (1–2 cm from stapled edge) and thus helped establishing clear boundaries of a true watershed segment.

### Perfusion differences in antimesenteric and mesenteric bowel regions

To further determine spatial sensitivity distinguishing tissue perfusion, we measured tissue perfusion between antimesenteric and mesenteric borders of the bowel across the induced gradient of bowel ischemia. For the perfused bowel segment, mean RPU over the mesenteric arc was 100% ± 3.3% while mean RPU over the antimesenteric arc was 83.4% ± 5.4%. In the perfused and watershed segments, RPU measurements showed an overall trend of increased perfusion to the mesenteric side of the bowel compared to the antimesenteric side (*p* < 0.00001 in perfused/watershed regions) (Fig. 3b).

### Differences in spatiotemporal accuracy between LSCI and ICG

**Fig. 4:**
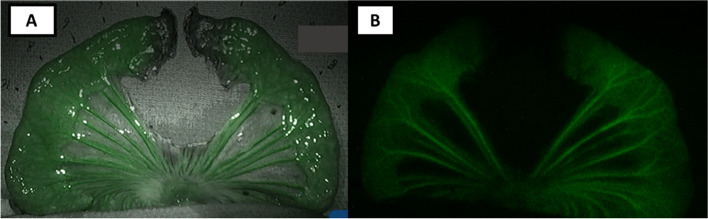
ICG detection in the linear small bowel model. Indocyanine green dye (0.1–0.3 mg/kg) was administered and the progressively de-vascularized small bowel ischemia gradient model was observed under ICG grayscale and ICG contrast mode at 30–40 s after injection. **A** shows ICG grayscale image of the small bowel gradient. **B** shows ICG contrast image of the small bowel gradient. Based on qualitative ICG images alone, a continuous gradient of bowel ischemia cannot be identified. Rather regions can be demarcated as perfused (green) 3–10 cm from stapled edge, not perfused (not green) 0–1 cm from stapled edge and suboptimal perfusion (lighter green) 1–3 cm from stapled edge

We then compared the real time display of tissue perfusion over the small bowel gradient using LSCI and ICG-FA. A standard ICG dose of 0.1–0.3 mg/kg was administered, and the bowel was observed approximately 30–40 s after injection, consistent with previous studies [15, 23, 25]. Rather than the continuous perfusion gradient seen on the LSCI colormap, visual inspection of the ICG modes could only qualitatively demarcate three different regions of perfusion over the bowel loop. Images were assessed by three surgeons and classified qualitatively as high intensity, low intensity, and no visible ICG signal. Regions with high ICG fluorescence signal intensity were considered perfused (3–10 cm from stapled edge), regions with no ICG signal fluorescence were considered non-perfused (0–1 cm from stapled edge) and regions with lower ICG fluorescence intensity were consider sub-optimally perfused (1–3 cm from stapled edge) (Fig. 4a, b).

### Differences in perfusion of bowel gradient with arterial and venous occlusion

**Fig. 5:**
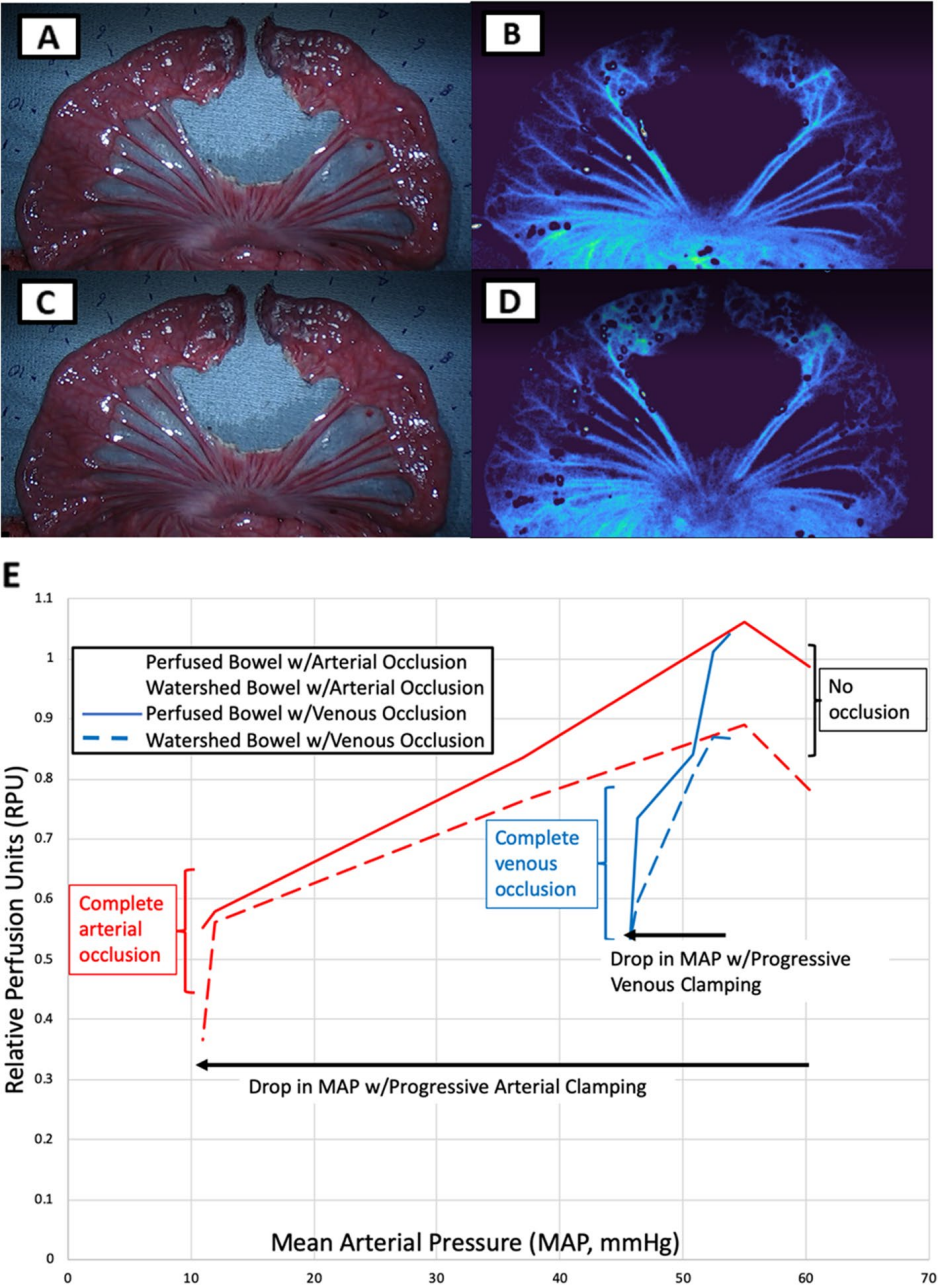
**a**-**d** Perfusion differences with arterial/venous occlusion of small bowel model. Arterial occlusion is achieved via clamping of proximal aorta and venous occlusion is achieved via clamping of portal vein. 5A RGB image of complete arterial occlusion of small bowel gradient model. 5B LSCI contrast image of complete arterial occlusion of small bowel gradient model. 5C RGB image of complete venous occlusion of small bowel gradient model. 5D LSCI contrast image of complete venous occlusion of small bowel gradient model. While there are no perfusion differences detected under visual inspection on RGB (5A and 5C), both arterial and venous occlusion result in ischemia that can be detected via the dark blue colormap on LSCI. Complete arterial and venous occlusion result in a similar low perfusion state that can be both observed and quantified using LSCI. **e** Effect of changes in mean arterial pressure via progressive arterial/venous occlusion) on bowel perfusion (measured in RPU). Relative Perfusion Units (RPU) can be seen on Y axis vs Mean Arterial Pressure (MAP, mm Hg) on X axis. The MAP gradient was created via progressive arterial occlusion and venous occlusion and MAP was measured downstream via arterial line in the iliac artery. Red lines indicate changes occurring resulting from progressive arterial occlusion in both perfused and watershed bowel, while blue lines indicate changes in both perfused and watershed bowel resulting from progressive venous occlusion. Solid lines represent the behavior of the perfused segment of bowel, while dashed lines represent the behavior of the watershed bowel segment. With both progressive arterial and venous occlusion, perfused/watershed segment RPUs had a strong linear relationship with MAP – higher MAPs in observed tissue correlated with higher RPU values, while lower MAPs induced by progressive clamp correlated with lower RPU values. For arterial occlusion (red lines), *R*^2^ = 0.96/0.79 for perfused/watershed bowel (bold/dash);for venous (blue lines) occlusion, *R*^2^ = 0.06/0.96 for perfused/watershed bowel (bold/dash). An increased sensitivity to venous occlusion was observed—small decreases in MAP secondary to venous occlusion resulted in large drops in mean RPU of bowel segments

To determine the temporal sensitivity of both colormap and RPU, the bowel gradient was observed under conditions of progressively arterial inflow and venous outflow occlusion. LSCI was able to detect and display changes in perfusion colormap and perfusion units resulting from ischemia secondary to arterial inflow and venous outflow occlusion (Fig. 5a-d). Both progressive arterial and venous occlusion induced a decline in Mean Arterial Pressure (MAP) and a concordant decrease in mean perfusion values over the perfused, watershed and ischemic bowel segments. With arterial inflow occlusion, mean RPU in perfused/watershed bowel segments demonstrated a strong linear correlation to MAP changes (*R*^2^ = 0.96/0.79 for perfused/watershed bowel). Similarly with venous outflow occlusion, mean RPU in perfused/watershed bowel segments demonstrated a strong linear correlation to MAP changes (*R*^2^ = 0.86/0.96 for perfused/watershed bowel) (Fig. 5e). Interestingly, LSCI showed a high sensitivity to venous occlusion, with small changes in MAP resulting in corresponding large changes in RPU.

### Differences in perfusion along side-to-side mesenteric vs antimesenteric anastomoses

**Fig. 6:**
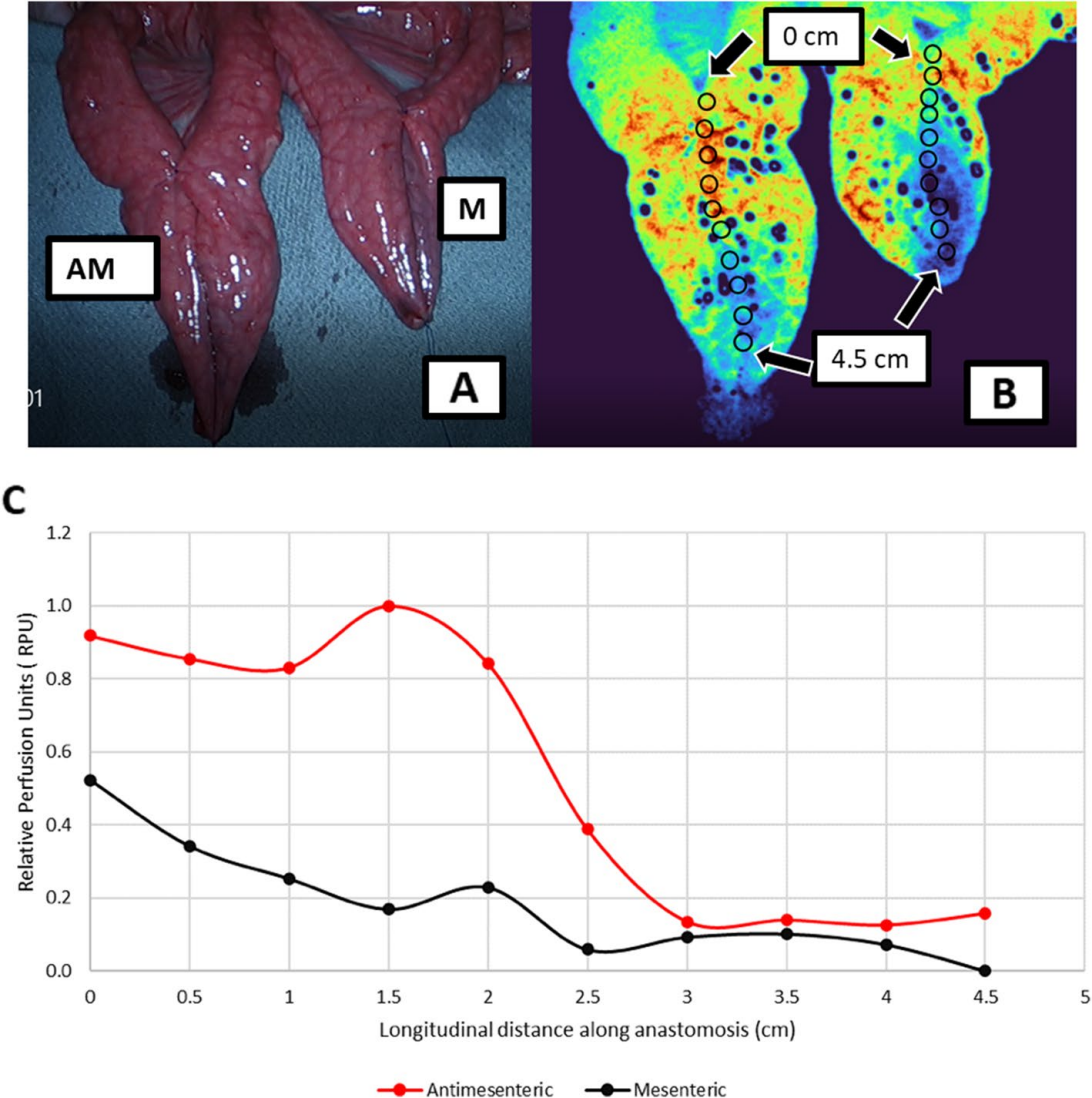
**a-b** Perfusion differences between antimesenteric and mesenteric anastomoses. Two randomly selected loops of small bowel were stapled to create side-to-side anastomoses, one on the mesenteric side and one on the antimesenteric side. 6A shows RGB image of the experimental setup with the porcine small bowel anastomoses models, antimesenteric anastomosis on left is labelled as AM, mesenteric anastomosis on right is labelled as M. 6B shows the experimental setup in LSCI contrast image. RPU’s were measured longitudinally along both anastomoses at the matched points labelled by the black circles (10 points starting at 0 cm to 4.5 cm). As compared to RGB image, the LSCI colormap shows increased perfusion to both anastomoses proximally (red/orange) as compared to distally (blue). Based on qualitative assessment alone, the mesenteric anastomosis (right) appears to have a higher concentration of blue/green regions than the antimesenteric anastomosis (left), indicating lower perfusion. **c** Differences in perfusion between mesenteric vs antimesenteric anastomoses. Distribution of Relative Perfusion Units (RPU) of antimesenteric and mesenteric small bowel anastomoses are shown on Y-axis as a function of distance (cm) along the anastomoses on X axis. Mesenteric perfusion is marked by solid red line whereas antimesenteric perfusion is marked by solid black line. The mean RPU of antimesenteric and mesenteric bowel was significantly different (*p* < 0.05), with antimesenteric measurements greater than mesenteric perfusion at every point measured along the anastomoses

To further evaluate potential differences in bowel perfusion not only within the same linear bowel setting with radial mesenteric blood supply, but in clinically relevant models such as intestinal anastomoses, two side-to-side small bowel anastomoses on the mesenteric-to-mesenteric and antimesenteric-to-antimesenteric border were created and examined under LSCI mode (Fig. 6a, b). RPU’s were measured longitudinally along both anastomoses at 10 matched points starting proximally at 0 cm to 4.5 cm distally. LSCI detected a significant difference in tissue perfusion between antimesenteric and mesenteric anastomoses (*p* = 0.002). For each set of points along the anastomoses, RPU measurements showed an overall trend of increased perfusion to the antimesenteric anastomosis as compared to the mesenteric anastomosis (Fig. 6c). The mean RPU along the antimesenteric anastomosis was 54% ± 18% while mean RPU along the mesenteric anastomosis was 38% ± 15%.

## Discussion

This preclinical study demonstrates that LSCI with a prototype quantification feature can detect and display real-time tissue perfusion in both color heatmaps as well as numerical units. LSCI detects a continuous gradient of perfusion linearly as a function of distance and can distinguishes among traditionally categorized ischemic, watershed and perfused regions of bowel based on naked eye inspection. LSCI also detects dynamic changes in tissue perfusion in real time resulting from arterial inflow and venous outflow obstructions. The spatiotemporal accuracy of LSCI demonstrates regional differences in perfusion between mesenteric and antimesenteric bowel. In the linearly de-vascularized small bowel model, the mesenteric region of the bowel had expectantly higher baseline perfusion compared to the antimesenteric side. When comparing anastomoses, LSCI detected significantly higher perfusion at the anastomosis made conventionally along the antimesenteric border compared to mesenteric border.

Novel techniques to assess real time bowel perfusion are gaining clinical utility because conventional methods to measure anastomotic perfusion have been found to be unreliable [26]. Recently, there has been wide adoption of ICG-FA for bowel perfusion assessment in colorectal surgery [27]. While efforts to quantify fluorescence intensity of ICG-FA are being studied [5], in its current widely adopted form, there is still subjectivity regarding the level of ICG fluorescence [28]. This may be because no consensus exists on the method of quantification of the ICG fluorescence, and no threshold for adequate perfusion has yet been identified [29]. Additionally, the studies that have described ICG-FA quantification efforts perform that analysis retrospectively and do not have it available in real time for clinical use intra-operatively [30]. In addition to its subjectivity, ICG-FA requires timed intravenous dye injection, and it is not amenable to repeat angiography due to dye diffusion and tissue infiltration [31, 32]. LSCI on the other hand does not require dye injection, is easily repeatable, has less latency to perfusion display compared to ICG [16].

In contrast to ICG-FA, LSCI with investigational perfusion quantification can quantify real time bowel perfusion and display it as both a colormap and a numeric value. Correlating the color on the perfusion heatmap with a numeric value allows for more objective and standardized assessment of bowel perfusion. With this study we establish that LSCI can detect a continuous gradient of bowel perfusion as a function of distance. Conventionally, regions of perfused, watershed and ischemic bowel in a de-vascularized bowel segment are determined via manual naked eye assessments. With LSCI, using relative perfusion metrics, we were able to identify that the bowel perfusion values started to sharply decline one cm away from the edge of mesenteric devascularization (5 cm from the stapled bowel edge). With this spatiotemporally accurate understanding of bowel perfusion, LSCI could help determine the true boundaries of the watershed region and support surgeon decision making with regards to preserving bowel length and defining the exact limits of resection. Further studies are needed to determine more precise RPU cut-off values for these intestinal regions and how these values translate into clinically relevant perfused or ischemic segments and subsequent risks for anastomotic leak.

Other preclinical studies that have used orthogonal polarization spectral imaging have been unable to detect significant regional differences in tissue perfusion between the anastomoses made on the antimesenteric vs the mesenteric border of the bowel [33]. Conventional techniques of side-to-side anastomosis creation recommend antimesenteric anastomosis as it avoids manipulation of the mesentery and thus does not interfere with blood supply [34]. This study confirms this conventional ‘wisdom’ because despite mesenteric perfusion being greater than antimesenteric perfusion in a linear small bowel loop, the antimesenteric anastomosis had higher perfusion than the mesenteric anastomosis. Thus, this technology can detect regional differences with accuracy and provide real time guidance in anastomosis creation.

The failure of anastomotic healing can be related to many surgical factors (perfusion and tension on the anastomosis) as well as patient factors such as co-morbidities and differences in intestinal microbiota [35]. The ability to distinguish between venous and arterial causes of ischemia may play an important role in understanding the surgical etiology of anastomotic complications [36, 37]. The ability to intra-operatively detect tissue ischemia and discriminate between arterial insufficiency or venous congestion could impact surgical strategy in colon resections. Venous congestion can cause circulatory disturbance, resulting in mucosal damage, ulceration or poor healing, followed by anastomotic leakage [38]. Other areas of surgery where distinction might be of use is in the construction of gastric conduit for esophagectomies or in reconstructive and graft surgery [39].

Although the device used in this study is FDA-cleared for clinical use, the generalizability of porcine anatomy and physiology to humans is assumed but requires further validation [40]. Additionally, LSCI perfusion detection is limited both by motion artifact as well as depth penetration [41]. This study was performed using a laparoscopic scope holder and at a standard distance of 20 cm in order to control distance and motion artifact, which have a known impact on LSCI accuracy. Twenty cm was chosen as the optimal distance in this study to ensure that the entire experimental model would be contained in the field of view, while also reducing angle artifact at the edges. The technology is designed and used in a laparoscopic form factor and can be used both intracorporeally and extracorporeally. Our next steps are to investigate and validate the impact of distance and angle on perfusion quantification in vivo in human trials.

In an in vivo study by Eriksson et. Al, artifacts caused by the heart beating accounted for 40% of their signal and respiratory artifacts showed up as low-frequency oscillations, which seemed to be a recurring theme in perfusion measurements on visceral organs [42]. In order to reduce motion artifact, some studies have applied post-hoc correction of measurements to compensate for heartbeat and respiration [43, 44]. In this study, relative perfusion values (that were obtained in real time) were then averaged over a cardiac cycle post-hoc in order to account for these artifacts. While these processing methods can account for signal artifacts post-operatively, there is still a need for robust real time effective motion artifact correction.

## Conclusion

This LSCI-enabled investigational perfusion algorithm has potential clinical utility for standardized and objective real time perfusion assessment of gastrointestinal anastomoses. In this preclinical study, we demonstrated that LSCI can quantify regional differences in bowel perfusion with spatiotemporal accuracy and detect real time changes in tissue perfusion induced by physiological shifts such as arterial and venous occlusion.

## Data Availability

The datasets used and/or analysed during the current study available from the corresponding author on reasonable request.
